# Twelve-Month Results From the First-in-China Prospective, Multi-Center, Randomized, Controlled Study of the FREEWAY Paclitaxel-Coated Balloon for Femoropopliteal Treatment

**DOI:** 10.3389/fcvm.2021.686267

**Published:** 2021-09-10

**Authors:** Bihui Zhang, Min Yang, Tao He, Xuan Li, Jianping Gu, Xiaoming Zhang, Xiangchen Dai, Xuedong Li, Xinwu Lu, Dehai Lang, Hongyao Hu, Xueming Chen, Baozhong Yang, Hongbin Gu, Xiwei Zhang, Yinghua Zou

**Affiliations:** ^1^Department of Interventional Radiology and Vascular Surgery, Peking University First Hospital, Beijing, China; ^2^Department of Vascular Surgery, The Central Hospital of Wuhan, Wuhan, China; ^3^Department of Interventional Radiology and Vascular Surgery, Peking University Third Hospital, Beijing, China; ^4^Department of Interventional Radiology and Vascular Surgery, Nanjing First Hospital, Nanjing, China; ^5^Department of Vascular Surgery, Peking University People's Hospital, Beijing, China; ^6^Department of Vascular Surgery, Tianjin Medical University General Hospital, Tianjin, China; ^7^Department of Vascular Surgery, The Second Hospital of Tianjin Medical University, Tianjin, China; ^8^Department of Vascular Surgery, Shanghai Ninth People's Hospital, Shanghai JiaoTong University School of Medicine, Shanghai, China; ^9^Department of Vascular Surgery, Ningbo No. 2 Hospital, Ningbo, China; ^10^Department of Vascular Surgery, Renmin Hospital of Wuhan University, Hubei General Hospital, Wuhan, China; ^11^Department of Vascular Surgery, Beijing Friendship Hospital, Capital Medical University, Beijing, China; ^12^Peripheral Vascular Department, Beijing University of Chinese Medicine Dongfang Hospital, Beijing, China; ^13^Department of Vascular Surgery, People's Liberation Army (PLA) Strategic Support Force Characteristic Medical Center, Beijing, China; ^14^Department of Vascular Surgery, Jiangsu Province Hospital, Nanjing, China

**Keywords:** drug-coated balloon, femoropopliteal lesions, randomized controlled trials, target lesion revascularization, FREEWAY

## Abstract

**Background:** Several paclitaxel-coated balloons have been proved to provide better efficacy results than uncoated balloons in femoropopliteal lesions. But the efficacy and safety of FREEWAY balloons have not been investigated in Chinese patients. This study aimed to evaluate the efficacy and safety performance of FREEWAY paclitaxel-coated balloons vs. uncoated balloons in Chinese femoropopliteal artery lesions.

**Methods:** In this prospective multi-center randomized controlled FREEWAY-CHINA study, 311 patients with symptomatic lower limb ischemia (Rutherford category 2–5) and femoropopliteal lesions of 14 Chinese centers were randomly assigned in a 1:1 ratio to endovascular treatment with either FREEWAY paclitaxel-coated balloons or uncoated balloons (control). The primary endpoint was the 6-month clinically-driven target lesion revascularization (CD-TLR) rate. Secondary endpoints included the device and technical success rate, the ankle-brachial indexes (ABIs), Rutherford category change, the 6-month primary and secondary patency rates, severe adverse effects, and the 12-month CD-TLR rate.

**Results:** The two groups were comparable in terms of their demographic and lesion characteristics. Patients' mean age was 70 years, and 70% were men. The mean lesion length was 71 mm. The 6-month CD-TLR rate was 2.6% in the FREEWAY group and 11.7% in the control group (*P* = 0.001). The 12-month CD-TLR rate was 2.7% in the FREEWAY group and 13.2% in the control group (*P* = 0.0005). Other endpoints, including patency rates, major adverse events, and ABI or Rutherford change, did not differ between the two groups.

**Conclusion:** The FREEWAY balloon resulted in an effective decrease in CD-TLR rates and had similar safety results compared to the uncoated balloon in Chinese femoropopliteal artery patients at the 12-month follow-up appointment.

## Introduction

Peripheral artery disease (PAD) is the third leading cause of atherosclerotic cardiovascular morbidity and has become a global problem (1). Endovascular treatment has been increasingly performed in clinical practice worldwide and is recommended for more complex lesions (2–6). Percutaneous transluminal angioplasty (PTA) has an initial effect in restoring blood flow but is limited by vessel recoil, remodeling, and intimal hyperplasia (7). Stents have improved patency and technical success, but the patency rates of 12 months in lesions longer than 10 cm remain poor, ranging from 50 to 65% (8–10). Concerns about stent fractures and intractable in-stent restenosis lesions also exist (11, 12).

The drug-coated devices, including drug-coated balloon (DCB) and drug-eluting stents (DES), are emerging therapeutic methods that multiple randomized controlled trials have shown promising results at reducing restenosis, target lesion revascularization, and late lumen loss (13–19). Although paclitaxel-related mortality and amputation raised concerns in recent years (20, 21), paclitaxel-coated devices are not associated with increased mortality in multiple researches (22–25). DCBs vary in terms of the materials used to make the balloons, the coating techniques, the choice of the coating drugs, and the drug's release patterns at the site (26). Additionally, patients of different races or ethnicities may react differently to the same therapy (27). The efficacy and safety of the new DCB-FREEWAY balloon in China in the context of precise medicine needed to be examined. Thus, the FREEWAY-CHINA study (a prospective, multi-center, randomized controlled trial on the FREEWAY paclitaxel-coated balloon's safety and efficacy vs. the conventional uncoated balloon in the treatment of femoropopliteal artery lesions in China) sought to investigate the performance of the FREEWAY DCB in Chinese femoropopliteal patients.

## Materials and Methods

### Study Design

The FREEWAY-CHINA study is a prospective, multi-center, randomized, controlled, superiority trial conducted in 14 hospitals in China (see Supplementary Appendix for details). This study aimed to evaluate the safety and efficacy of the FREEWAY paclitaxel-coated balloon (EUROCOR GmbH, Bonn, Germany) in *de novo* and restenotic femoropopliteal artery lesions by comparing a FREEWAY DCB to an uncoated balloon (JOKER balloon, EUROCOR GmbH, Bonn, Germany). The coating was paclitaxel (3 mg/mm^2^), and the shellac was applied as excipient using a micro-pipetting procedure. Shellac is a natural resin composed of shellolic and alleuritic acid used to coat gastric-resistant tablets and is European Medicines Agency (EMA) and US Food and Drug Administration (FDA) approved and generally recognized as safe. Patients with successful pre-dilation were randomly assigned using a 1:1 ratio to either the FREEWAY DCB treatment group or the uncoated balloon control group by a central randomization computer system. Patients with unsuccessful pre-dilation were considered screening failures and were not included in the study.

### Ethics

The study involving human participants was reviewed and approved by the Ethical Committees of the Peking University First Hospital and other participating centers. Patients provided written informed consent before enrolment. The trial was conducted following the Declaration of Helsinki and the provisions for the conduct of clinical trials of medical devices issued by the China Food and Drug Administration.

### Patient Population

Patients eligible for inclusion in the trial had intermittent claudication or critical limb ischemia (Rutherford category 2–5) and an angiographically significant atherosclerotic lesion (diameter of stenosis ≥ 70%) in the superficial femoral or popliteal artery (or both). The total treated lesion length had to be 15 cm or less, and the reference diameter of the target vessel had to be 3–8 mm. Successful guidewire crossing and pre-dilation were acquired. Patent or successfully recanalized inflow vessels and at least one outflow vessel was needed.

Patients were excluded from the study if they met any of the following exclusion criteria: had severe calcification of the target vessel; had previously undergone surgery of the target femoropopliteal segment; had acute or subacute thrombosis or embolization of the target vessel; and/or had undergone adjuvant therapy (e.g., laser, directional or rotational atherectomy, cryoplasty, or scoring/cutting balloon). The complete inclusion and exclusion criteria are set out in the Supplementary Appendix.

### Procedure and Medication

**Figure 1:**
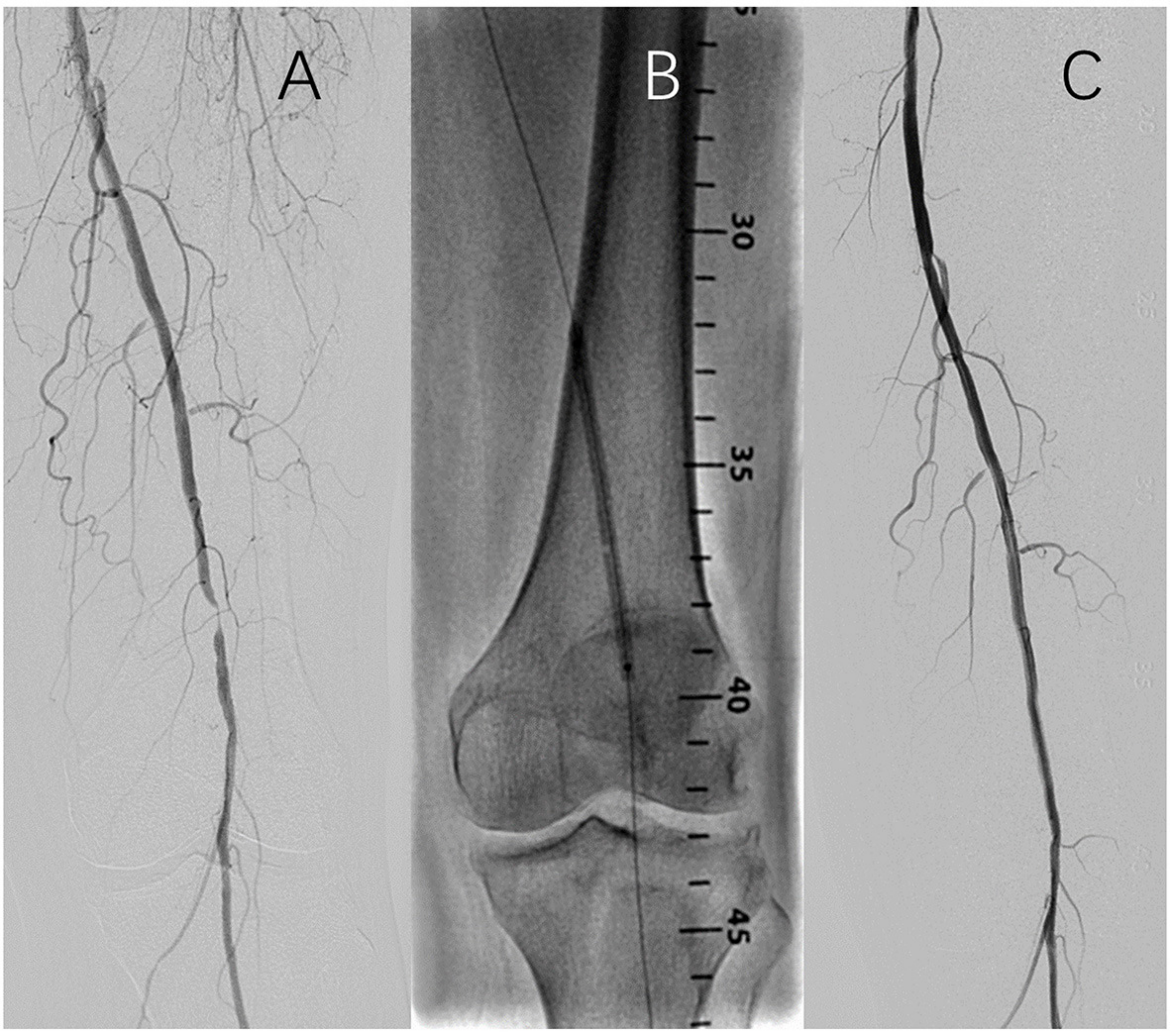
Representitive case of the endovascular procedure. **(A)** Baseline angiography; **(B)** Balloon inflation; **(C)** Completion angiography.

The target lesion was pre-dilated with a balloon/vessel ratio of 0.9:1–1:1 after successful lesion crossing during the operation. A FREEWAY DCB or a Joker PTA balloon was used according to the results of the randomization. For DCB sizing, the nominal balloon diameter had to match the reference vessel diameter distal to the target lesion. To secure full lesion coverage, the DCB or uncoated balloon's length was required to cover at least 1 cm proximal and distal to the lesion. DCB inflation time should be at least 180 s for the first dilation. In case of residual stenosis of >30% after the first balloon dilation, the same type of balloon was used for the second dilation for > 120 s. A new FREEWAY DCB had to be used in the FREEWAY group; however, the same Joker uncoated balloon could be used in the control group. After the second dilation, no treatment was needed if the residual stenosis was >30% but <50%. Laser-cut self-expanding nitinol stents had to be implanted when residual stenosis was >50% or D-type dissection was found after the second dilation, and covered stents were not allowed. If two or more DCBs were needed, the overlap had to be at least 0.5 cm. Each lesion was covered by 2 DCBs at most. A representative case is shown in Figure 1.

Clopidogrel was given for at least 3 days (75 mg/day) before or immediately after the intervention (300 mg). Heparin (3,000–5,000 units) was given intravenously during the procedure. After the procedure, clopidogrel (75 mg/day) was recommended for 4 weeks. Patients were given aspirin (100 mg daily/long-term) before and after endovascular treatment.

### Follow-Up

A 30-day follow-up appointment was conducted by phone or in-house to evaluate patients' clinical status, medication compliance, and adverse events. Concerning patients' in-house follow-up clinical status, the calculation of ankle-brachial indexes (ABIs) at rest, duplex ultrasonography, and medicine compliance were performed after the procedure and at 6 and 12 months.

### Endpoints and Definitions

The primary endpoint was the clinically-driven target lesion revascularization (CD-TLR) rate at the 6-month follow-up appointment. The term “clinically driven” was defined as an increase in the Rutherford grade and a Doppler ultrasound scan showing diameter stenosis of ≥ 70%. Revascularization included arterial endarterectomy, bypass grafting, or an endovascular procedure.

The secondary endpoints included: (1) Device success rate, which was defined as the percentage of successful balloon placement and expansion without rupture and successful withdrawal; (2) Technical success rate, which was defined as a percentage of residual stenosis of ≤ 30% after target lesion treatment; (3) an ABI increased by more than 0.1 (the ABIs at discharge and the follow-up appointment compared to the ABI at baseline); (4) The primary and secondary patency rates at the 6-month follow-up appointment. Patency was defined as a peak systolic velocity of ≤ 2.4 m/s, as evaluated by duplex ultrasonography; (5) Major adverse events including all-cause death, revascularization, or major amputation; (6) Any changes in Rutherford classification from preoperative to discharge, 30 days, 6 months, and 12 months; (7) The incidence of CD-TLR at 12 months after the procedure; and (8) Device-associated severe adverse effects anytime in the process of the trial. Severe adverse events were defined as definite, probable, or possible device-associated events that required patient hospitalization, a prolonged hospitalization time, caused disability or death, affected workability, or led to congenital malformations.

### Statistical Analysis

This study was a superiority trial. It was assumed that the CD-TLR rate was 8% in the experimental group 6 months after the use of DCB, while the uncoated balloon's CD-TLR rate was 20% in the control group. The FREEWAY and control groups were allocated according to the ratio of 1:1, and each group had to comprise 131 patients (to achieve at least 80% power). Assuming a 15% attrition rate, a cohort of 312 patients was needed for the analysis.

The outcomes were analyzed using the full analysis set (FAS) according to the intention-to-treat principle. Categorical data were described by frequency and ratio. Continuous data were described as mean ± standard deviation, maximum, minimum, median, 25th, and 75th quantile. Chi-square tests were used to compare the categorical data between the groups. An independent *t*-test was used to compare the continuous data of normal distribution. A Wilcoxon rank-sum test was used to compare the continuous data of non-normal distribution. For the 6-month CD-TLR rates, the likelihood ratio Chi-square/Fisher exact test, the Cochran–Mantel–Haenszel (CMH) Chi-square test with an adjusted center effect, the Kaplan Meier method, and the Tipping Point Method were used. A P value of 95% confidence interval (CI) was given on the FAS, per-protocol set (PPS), and actual treatment set (ATS). For the other 6-month and 12-month event rates, the likelihood ratio Chi-square/Fisher exact test was used. Mortality and adverse effects analyses were performed on the safety set (SS). All statistical analyses were performed using SAS 9.3 (SAS Institute Inc., Cary, NY, USA) at a bilateral 0.05 significance level.

## Results

### Patient and Procedural Characteristics

**Figure 2:**
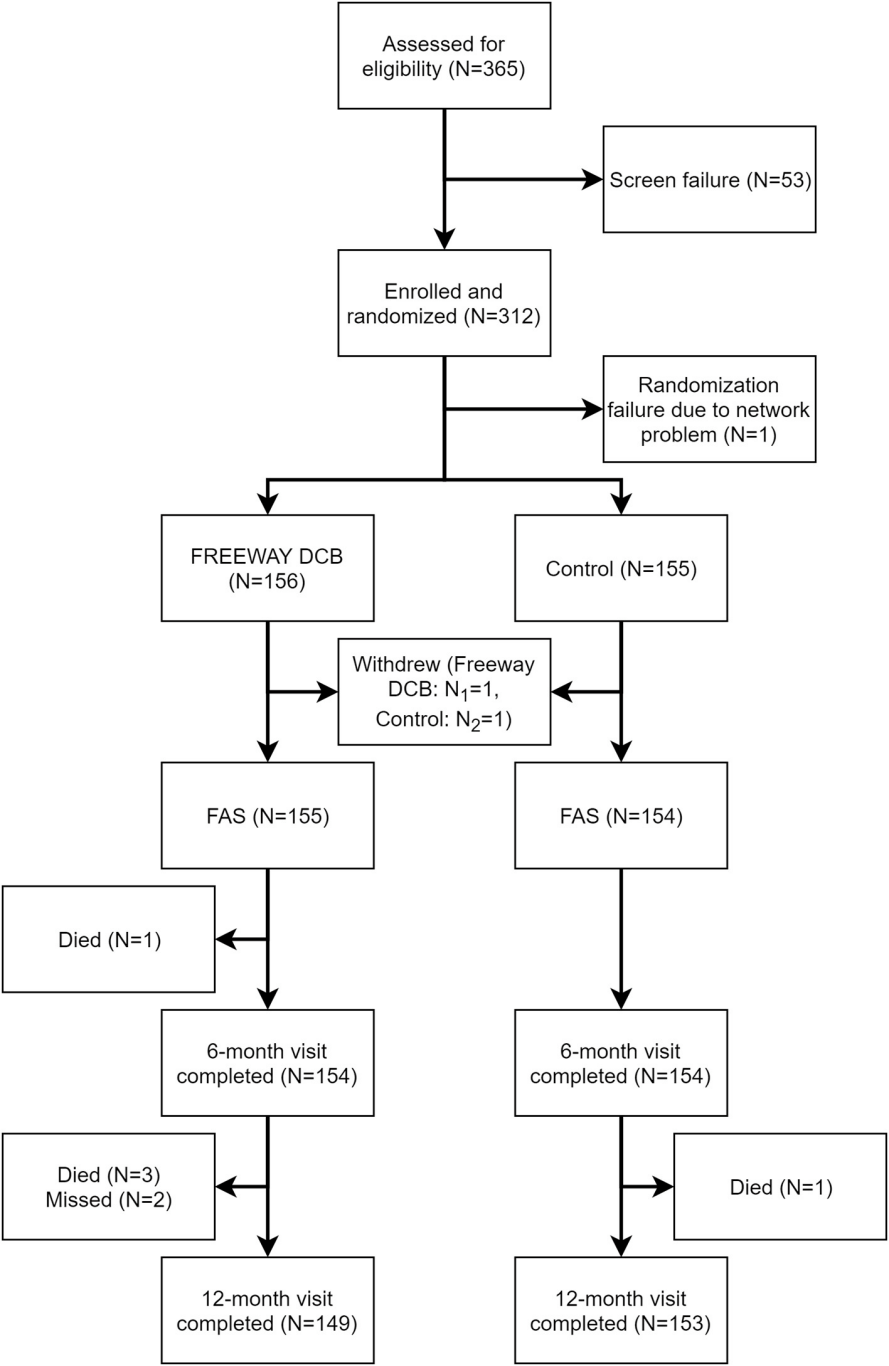
Patient flow in the FREEWAY-CHINA study. FAS, Full analysis set.

From July 9, 2015, to May 10, 2018, 311 patients (FREEWAY group:156, control group: 155) were enrolled at 14 centers in China, and the follow-up appointments were completed on May 4, 2019. Figure 2 shows the patient flowchart. The average age of patients was 69.87 ± 9.63 years old in the FREEWAY group and 70.70 ± 8.57 years old in the control group (*P* = 0.43). The proportion of males was 74.8% in the FREEWAY group and 66.2% in the control group (*P* = 0.10). The FREEWAY group had a higher proportion of diabetes (FREEWAY group: Type I 2.6%, Type II 62.6%, control group: Type I 0.6%, Type II 49.4%, *P* = 0.011). There was no significant difference between the two groups for the other risk factors, including hypertension, coronary heart disease, stroke, smoking, and renal insufficiency (see Table 1).

**Table 1:** Patient characteristics.

	**FREEWAY group (** ***N*** **= 155)**	**Control group (** ***N*** **= 154)**	***P***
Age	69.87 ± 9.63	70.7 ± 8.57	0.426
Male	116 (74.8)	102 (66.2)	0.097
Hypertension	108 (69.7)	121 (78.6)	0.074
Diabetes			0.011
Type I	4 (2.6)	1 (0.6)	
Type II	97 (62.6)	76 (49.4)	
Dyslipidemia	20 (12.9)	24 (15.6)	0.500
Coronary heart disease	49 (31.6)	43 (27.9)	0.478
Stroke	39 (25.1)	39 (25.3)	0.702
Renal insufficiency	2 (1.3)	1 (0.6)	1.000
Previous peripheral artery disease	55 (35.5)	64 (41.6)	0.272
Smoking	73 (47.1)	71 (46.1)	0.979
Rutherford category			0.657
2	34 (21.9)	27 (17.5)	
3	78 (50.3)	76 (49.4)	
4	25 (16.1)	28 (18.2)	
5	18 (11.6)	23 (14.9)	
Aspirin	115 (74.2)	113 (73.4)	0.870
Clopidogrel	106 (68.4)	100 (64.9)	0.520

*Data are reported as N (%) or mean ± standard deviation when appropriate*.

There were more cases of multiple lesions in the FREEWAY group (FREEWAY group: 1 lesion 83.9%, 2 lesions 14.2%, 3 lesions 1.9%, control group: 1 lesion 92.2%, 2 lesions 7.8%, 3 lesions 0%, *P* =0.03). There was no significant difference in reference vessel diameter, lesion length, and stenosis rate between the two groups. The pre-dilation balloon diameter was larger in the FREEWAY group than the control group (4.31 ± 0.69 mm vs. 4.13 ± 0.64 mm, *P* = 0.011). Second balloon dilation occurred in a higher proportion in the FREEWAY group (9.8 vs. 2.4%, *P* = 0.003). Bailout stenting after the second dilation was less frequent in the FREEWAY group than the control group(64.5 vs. 82.5%, *P* = 0.026); however, the overall bailout stent rate by patient-level did not differ between the groups (25.8 vs. 30.5%, *P* = 0.357). Details of the angiographic and procedural characteristics are shown in Table 2.

**Table 2:** Lesion and procedural characteristics.

	**FREEWAY group (** ***N*** **= 183)**	**Control group (** ***N*** **= 166)**	***P***
Right limb	84 (45.9)	86 (51.8)	0.270
Lesion location			0.307
SFA	168 (91.8)	147 (88.6)	
Popliteal	15 (8.2)	19 (11.4)	
Reference vessel diameter (mm)	4.87 ± 0.61	4.89 ± 0.59	0.770
Lesion length (mm)	69.9 ± 40.3	72.2 ± 40.3	0.599
Diameter stenosis (%)	91.7 ± 10.4	90.9 ± 10.5	0.457
Lesion type			0.730
*De novo*	161 (88.0)	148 (89.2)	
Restenotic	22 (12.0)	18 (10.8)	
Pre-dilation balloon diameter (mm)	4.31 ± 0.69	4.13 ± 0.64	0.011
Pre-dilation balloon length (mm)	102.4 ± 47.2	101.8 ± 47.3	0.913
Experimental balloon number^a^			0.003
1	165 (90.2)	162 (97.6)	
2	18 (9.8)	4 (2.4)	
Experimental balloon diameter (mm)	4.86 ± 0.55	4.86 ± 0.54	0.875
Experimental balloon length (mm)	103.9 ± 34.3	103.5 ± 32.6	0.920
Residual stenosis after experimental balloon dilation			0.970
≤ 30%	121 (66.1)	109 (65.7)	
>30%	50 (27.3)	45 (27.1)	
≤ 30% and further treatment	12 (6.6)	12 (7.2)	
Bailout stent number (lesion-level)			0.326
0	143 (78.1)	119 (71.7)	
1	37 (20.2)	42 (25.3)	
2	3 (1.6)	5 (3.0)	

*Data are reported as N (%) or mean ± standard deviation when appropriate*.

a *Experimental balloon was defined as FREEWAY or JOKER balloon*.

### Endpoint Evaluation

The overall complication rate was similar between the 2 groups (27.1 vs. 26.0%, *P* = 0.823), most of which were dissections (23.2 vs. 18.8%, *P* = 0.343). Hematomas at the puncture occurred more frequently in the control group than the FREEWAY group (0 vs. 3.2%, *P* = 0.030). Other complications did not differ between the two groups (see Table 3). The device success rates were 100% in both groups, and the technical success rate did not differ between the groups (FREEWAY group 96.2%, control group 98.2%, *P* = 0.343).

**Table 3:** Complications in the procedures.

	**FREEWAY group (** ***N*** **= 155)**	**Control group (** ***N*** **= 154)**	***P***
Overall	42 (27.1)	40 (26.0)	0.823
Vessel spasm	0 (0)	1 (0.6)	0.498
Dissection	36 (23.2)	29 (18.8)	0.074
Pseudoaneurysm	1 (0.6)	1 (0.6)	1.000
Puncture site hematoma	0 (0)	5 (3.2)	0.030
Other complications	8 (5.2)	7 (4.5)	0.801

*Data are reported as N (%)*.

The 6-month CD-TLR rate was 2.6% in the FREEWAY group and 11.7% in the control group on FAS (*P* = 0.001). The 95% CI of the incidence difference of 6-month CD-TLR (FREEWAY group vs. control group) was <0% when analyzed using various FAS, PPS, and ATS, which proved the superiority of the FREEWAY balloon (see Table 4). The 6-month primary patency rate of the FREEWAY group was 70.1%, and that of the control group was 62.3%; thus, the rate was slightly higher in the FREEWAY group, but the difference was not significant (*P* = 0.115). The 6-month secondary patency rates were similar between the 2 groups (71.4% in the FREEWAY group and 71.0% in the control group, *P* = 0.786). The 12-month CD-TLR rate was 2.7% in the FREEWAY group and 13.2% in the control group (*P* = 0.0005). The rates of major adverse events including all-cause death, revascularization, and major amputation occurred in similar proportions in both groups at 1, 6, and 12 months (see Table 5). Additionally, the device-associated severe adverse effects did not differ between the FREEWAY and control groups (6.4 vs. 9.7%, *P* = 0.288).

**Table 4:** The 6-month CD-TLR rate difference of two groups analyzed by different methods.

**Data set**	**Likelihood ratio Chi-square/Fisher exact test^a^**	**CMH Chi-square with adjusted center effect^b^**	**Kaplan Meier method^b^**	**Tipping point method^b^**
FAS	0.0013	−8.4% [−10.5%; −3.0%]	−9.1% [−14.8%; −3.4%]	−9.0% [−10.8%; −4.1%]
PPS	0.0023	−8.0% [−10.1%; −2.4%]	−8.7% [−14.4%; −3.0%]	−8.7% [−10.4%; −3.6%]
ATS	0.0022	−8.0% [−10.1%; −2.4%]	−8.7% [−14.4%; −3.0%]	−8.7% [−10.4%; −3.6%]

*Data are reported as N (%)*.

*CD-TLR, clinically driven target lesion revascularization; FAS, full analysis set; PPS: per-protocol analysis set; ATS, actual treatment set*.

a *P value*.

b *Incidence difference (FREEWAY group-control group) and 95% confidence interval*.

**Table 5:** Summary of major adverse events at follow up.

		**FREEWAY group**	**Control group**	***P***
30-day	Death^a^	0 (0)	0 (0)	NA
	Revascularization^b^	6 (3.9)	4 (2.6)	0.750
	Major amputation^b^	0 (0)	0 (0)	NA
6-month	Death	5 (3.2)	2 (1.3)	0.448
	Revascularization	24 (15.6)	27 (17.5)	0.646
	Major amputation	1 (0.6)	1 (0.6)	1.000
12-month	Death	7 (4.5)	4 (2.6)	0.348
	Revascularization	26 (17.4)	31 (20.3)	0.532
	Major amputation	1 (0.7)	1 (0.7)	1.000

*Data are reported as N (%)*.

*NA, Not applicable*.

a *Death is analyzed on a safety set (SS)*.

b *Revascularization and major amputation are analyzed on a full analysis set (FAS)*.

**Figure 3:**
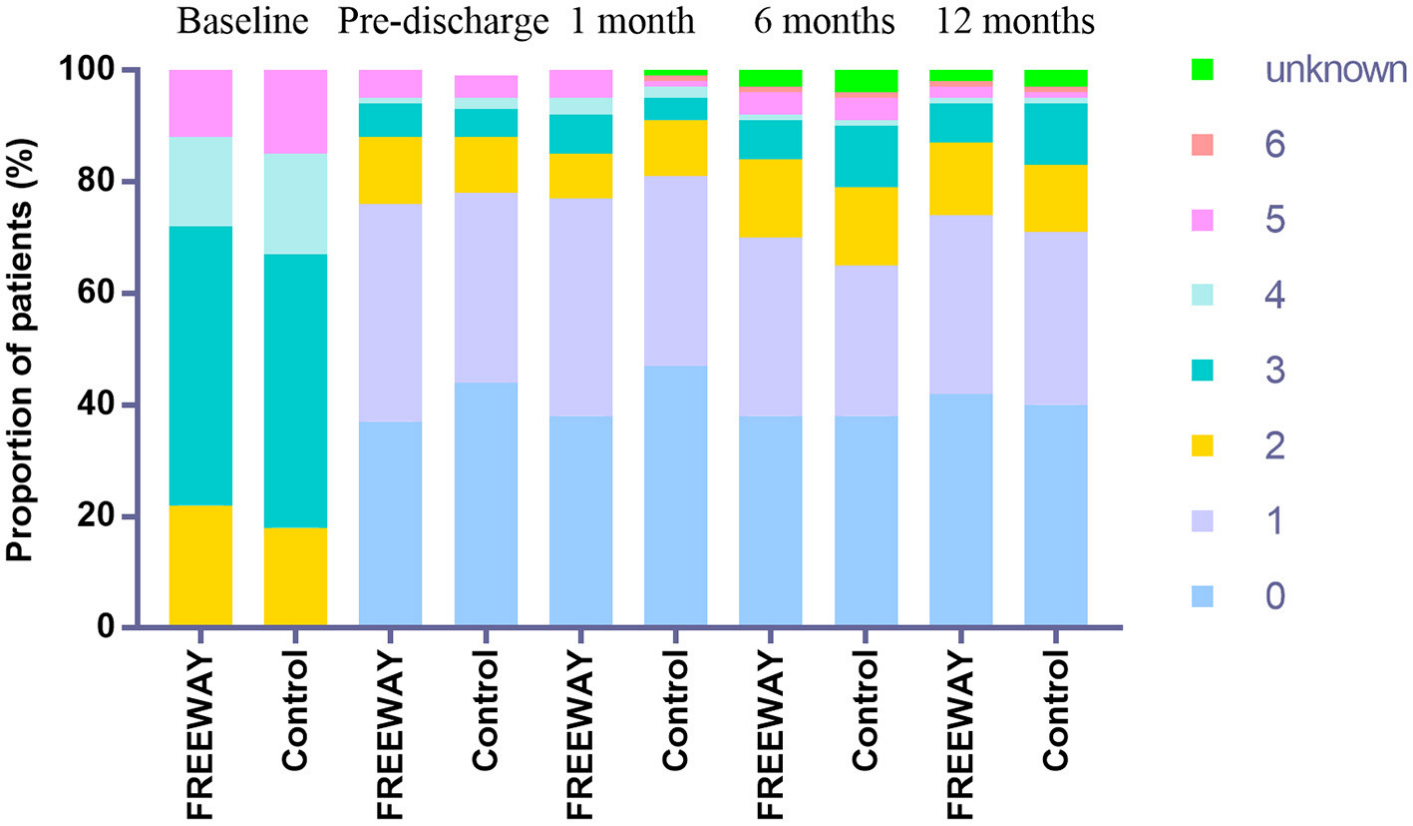
Rutherford category distribution at baseline, before discharge, at 1, 6, and 12 months.

The ABI elevation of the target limb by at least 0.1 compared with the baseline was 80.5% in the FREEWAY group and 78.2% in the control group before discharge (*P* = 0.651). At the 6-month follow-up time, the ABI elevation of the target limb by at least 0.1 compared with the baseline was 68.8% in the FREEWAY group and 59.5% in the control group (*P* = 0.141). The percentage of the decline in the Rutherford classification categories was similar between the two groups at 6 (FREEWAY group 81.8%, control group 79.2%, *P* = 0.844) and 12 months (FREEWAY group 86.6%, control group 84.1%, *P* = 0.856). The distribution of Rutherford categories at the baseline and follow-up times are shown in Figure 3.

## Discussion

The randomized controlled FREEWAY-CHINA study showed that the FREEWAY paclitaxel-coated balloon was superior to the uncoated balloon in terms of the 6- and 12-month CD-TLR rates in Chinese patients. Other efficacy endpoints, including 6-month primary/secondary patency rates, clinical status, and ABI, did not differ between the two groups. Safety endpoints, of the FREEWAY balloon, including all-cause death, revascularization, major amputation, and the device-associated severe adverse effects, were also similar between the FREEWAY and control groups.

Several randomized controlled trials have shown that paclitaxel-coated balloons have superior patency rates and freedom from CD-TLR rates than uncoated balloons (15, 16, 28–30). DCBs were shown to have higher primary patency (82.2 vs. 52.4%, *P* < 0.001) and lower CD-TLR rates (2.4 vs. 20.6%, *P* < 0.001) at 12 months in the IN. PACT SFA trial (16). Primary patency rates of 12 months were also shown to be higher in the DCB group than in the uncoated balloon group in the LEVENT 2 trial (65.2 vs. 52.6%, *P* = 0.02) (15). A low-dose paclitaxel-coated DCB was non-inferior to a high-dose paclitaxel-coated DCB concerning primary patency over 12 months in femoropopliteal interventions (31). The FREEWAY balloons provided better 12-month primary patency and freedom from CD-TLR rates than uncoated balloons in in-stent restenosis in femoropopliteal arteries in a PACUBA trial (32). The CD-TLR rates were also lower in the FREEWAY group than the control group in the present study (2.6 vs. 11.7% at 6 months, *P* = 0.001; 2.7 vs. 13.2%, *P* = 0.0005). Thus, the FREEWAY balloon could be another effective treatment option for DCBs for femoropopliteal patients.

Multiple trials have compared DCBs and uncoated balloons; however, most of the efficacy and safety results were obtained from Caucasian participants. As ethnicity may affect the pharmacological response to therapies, uncertainties about the use of paclitaxel-coated balloons in Chinese patients remain (27). Previously, the AcoArt I Trial was the only published randomized controlled trial to compare DCBs and uncoated balloons in 200 Chinese patients (28). Similarly, the target lesion revascularization rates were 7.2% in the Acotec DCB group and 39.6% in the control group (*p* < 0.001). The 6-month LLL and restenosis rate was also lower in the FREEWAY group in the AcoArt I Trial. The FREEWAY-CHINA trial results provide further evidence of the efficacy of paclitaxel-coated balloons in treating femoropopliteal lesions in Chinese patients.

The debate about paclitaxel's safety has been fierce since the publication of a meta-analysis showing higher 2- and 5-year all-cause mortality rates and heightened risk of major amputation in the paclitaxel group than control (20, 21). Although an association between increased all-cause mortality with paclitaxel was not found in large cohort studies and patient-level meta-analysis of randomized trials, concerns remain (22, 24, 33–37). In the present study, in which the follow-up rate was 98.7% in the FREEWAY group and 100% in the control group in the SS, the 12-month all-cause mortality and major amputation rates did not differ (*P* = 0.348 and *P* = 1.000). Thus, the FREEWAY balloon is safe for Chinese patients.

This study had several limitations. First, the endpoint evaluation was not performed by independent core laboratories. Second, patients with severe calcified and long lesions were not enrolled in this study, limiting the extrapolation. Third, long-term mortality data need to be examined further to prove FREEWAY balloons' safety in Chinese patients.

## Conclusions

In conclusion, in the multi-center, randomized controlled, FREEWAY-CHINA study, we demonstrated that the 6- and 12-month CD-TLR rates were lower in the FREEWAY group than the uncoated balloon group, and the safety results were similar between the FREEWAY group and the uncoated balloon group.

## Data Availability Statement

The raw data supporting the conclusions of this article will be made available by the authors, without undue reservation.

## Ethics Statement

The study involving human participants was reviewed and approved by Ethical Committees of the Peking University First Hospital, The Central Hospital of Wuhan, Peking University Third Hospital, Nanjing First Hospital, Peking University People's Hospital, Tianjin Medical University General Hospital, The Second Hospital of Tianjin Medical University, Shanghai Ninth People's Hospital, Ningbo No. 2 Hospital, Renmin Hospital of Wuhan University, Hubei General Hospital, Beijing Friendship Hospital, Beijing University of Chinese Medicine Dongfang Hospital, PLA Strategic Support Force Characteristic Medical Center and Jiangsu Province Hospital. The patients/participants provided their written informed consent to participate in this study. Written informed consent was obtained from the individual(s) for the publication of any potentially identifiable images or data included in this article.

## Author Contributions

BZ and MY contributed in writing the manuscript. YZ contributed in organizing, supervising, and revising. All the other authors contributed in organizing the study and revising the manuscript.

## Funding

This study was funded by Shanghai Micro Medical Devices.

## Conflict of Interest

The authors declare that this study received funding from Shanghai Micro Medical Devices. The funder was not involved in the study design, collection, analysis, interpretation of data, the writing of this article or the decision to submit it for publication.

## Publisher's Note

All claims expressed in this article are solely those of the authors and do not necessarily represent those of their affiliated organizations, or those of the publisher, the editors and the reviewers. Any product that may be evaluated in this article, or claim that may be made by its manufacturer, is not guaranteed or endorsed by the publisher.
