# Supplementary material for: Twelve-Month Results From the First-in-China Prospective, Multi-Center, Randomized, Controlled Study of the FREEWAY Paclitaxel-Coated Balloon for Femoropopliteal Treatment
Source: Front Cardiovasc Med. 2021 Sep 10;8:686267. doi: 10.3389/fcvm.2021.686267 (PMC8460758; doi:10.3389/fcvm.2021.686267)
Supplement: Supplementary file 1 [file Table_1.DOCX]

Participating hospitals and locations:

1. Department of Interventional Radiology and Vascular Surgery, Peking University First Hospital, Beijing, China

2. The Central Hospital of Wuhan, Wuhan, China

3. Peking University Third Hospital, Beijing, China

4. Nanjing First Hospital, Nanjing, China

5. Peking University People's Hospital, Beijing, China

6. Tianjin Medical University General Hospital, Tianjin, China

7. The Second Hospital of Tianjin Medical University, Tianjin, China

8. Shanghai Ninth People's Hospital, Shanghai JiaoTong University School of Medicine

9. Ningbo No. 2 Hospital, Shanghai, China

10. Renmin Hospital of Wuhan University, Hubei General Hospital, Wuhan, China

11. Beijing Friendship Hospital, Capital Medical University, Beijing, China

12. Beijing University of Chinese Medicine Dongfang Hospital, Beijing, China

13. PLA Strategic Support Force Characteristic Medical Center, Beijing, China

14. Jiangsu Province Hospital, Nanjing, China

Criteria:

Inclusion criteria:

Patients must meet the following criteria:

1. The subjects should have symptoms of lower limb ischemia (Rutherford category 2-5) and femoropopliteal arteries need to be treated;

2. Subjects must be male or non-pregnant female over 18 years old;

3. Single and/or multiple primary occlusive, stenotic, re-occlusive, or re-stenotic lesions in the femoral and/or popliteal arteries were included. The degree of stenosis should be more than 70% and the total length should be no longer than by visual assessment on angiography;

4. The diameter of reference target vessel was 3 mm-8 mm confirmed by angiography

5. The outflow should be patent or successfully recanalized (stenosis in diameter < 50%)

6. Successful wire crossing and pre-dilation of the target lesion;

7. The target lesion can be covered by two FREEWAY balloons

8. The stenosis of inflow artery should be less than 50%;

9. The FREEWAY balloon is suitable for treatment at the researchers’ discretion.

10. Subjects are willing to provide signed informed consent, comply with the research requirements, and accept the prescribed clinical follow-up and examinations.

Exclusion criteria:

Patients who meet any of the following criteria should be excluded:

1. Subjects who are pregnant or plan to get pregnant in the next year.

2. Subjects who have undergone surgical or interventional treatment of ipsilateral artery in the past 30 days before enrollment or plan to have surgical or interventional treatment of ipsilateral artery within 30 days after the index procedure.

3. Subjects whose Rutherford category are difficult to evaluate.

4. Presence of aneurysms in abdominal aorta, iliac artery or femoropopliteal artery.

5. Any coagulation disorders that prohibit antiplatelet therapy.

6. Intolerance to the drugs and contrast agents used in the study.

7. Presence of liver or kidney function damage, not suitable for interventional therapy.

8. Acute or subacute thrombosis or embolism of target vessels.

9. Absence of normal artery segment at the proximal end of the lesion for Doppler ultrasound to measure the ratio of blood flow velocity.

10. Presence of heavily calcified lesions.

11. Use of adjuvant therapies (laser, atherectomy, cryoplasty, scoring / cutting balloon, etc.).

12. Target lesion of bridge vascular in previous bypass operation;
